# Supplementary material for: Green Synthesis of Silver Nanoparticles Using Paullinia cupana Kunth Leaf Extract Collected in Different Seasons: Biological Studies and Catalytic Properties
Source: Pharmaceutics. 2025 Mar 10;17(3):356. doi: 10.3390/pharmaceutics17030356 (PMC11945093; doi:10.3390/pharmaceutics17030356)

Supporting Information

Supplementary Figure S1: Distribution histogram and 3D graphs of concentration/intensity *versus* hydrodynamic diameter of (A) AgNPs-LD and (B) AgNPs-LR.

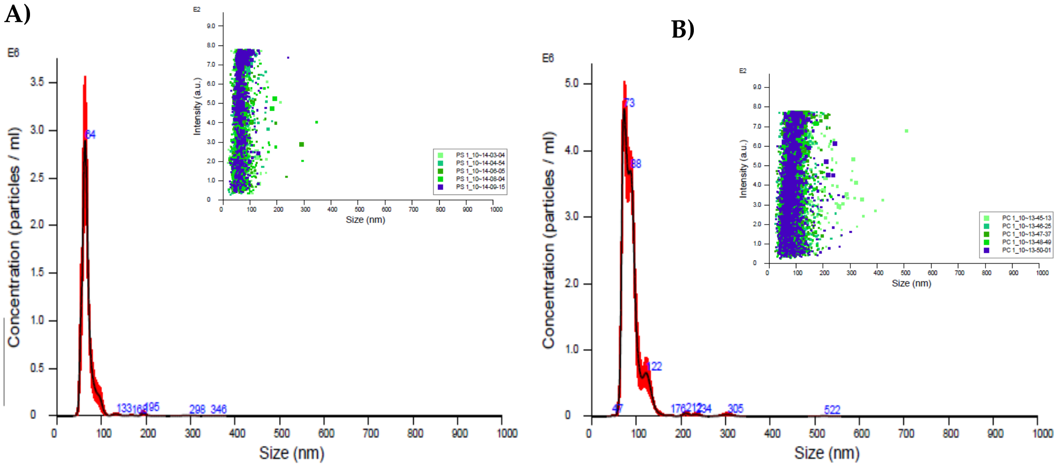

Supplementary Table S1. Cytotoxic effects of AgNPs, *Paullinia cupana* plant extracts and aqueous $AgNO_3$ solution expressed as IC_{50} values.

Samples	IC_{50} (µg/mL) – 24 h			
	**A431**	**HaCaT**	**A549**	**HNTMC**
**$AgNO_3$**	5.122	3.497	4.750	3.490
**AgNPs-LD**	5.851	3.940	5.436	4.000
**AgNPs-LR**	5.510	3.909	5.609	4.231
**Ext-LD**	458.2	1106	827.5	998.3
**Ext-LR**	638.1	1052	1017	309.6

Supplementary Figure S2: Antipromastigote activity of AgNPs, *Paullinia cupana* leaf extracts, aqueous $AgNO_3$ solution and miltefosine against *Leishmania amazonensis* after 72 h of incubation. The graph represents the mean ± standard deviation of the mean of four independent experiments, considering the control (0.5% DMSO in complete Schneider medium) as 0% inhibition. One-way ANOVA followed by the Bonferroni post-test was carried out to compare the groups, with *p<0.05; **p<0.01; ***p<0.001 and **** p<0.0001 compared to the control.

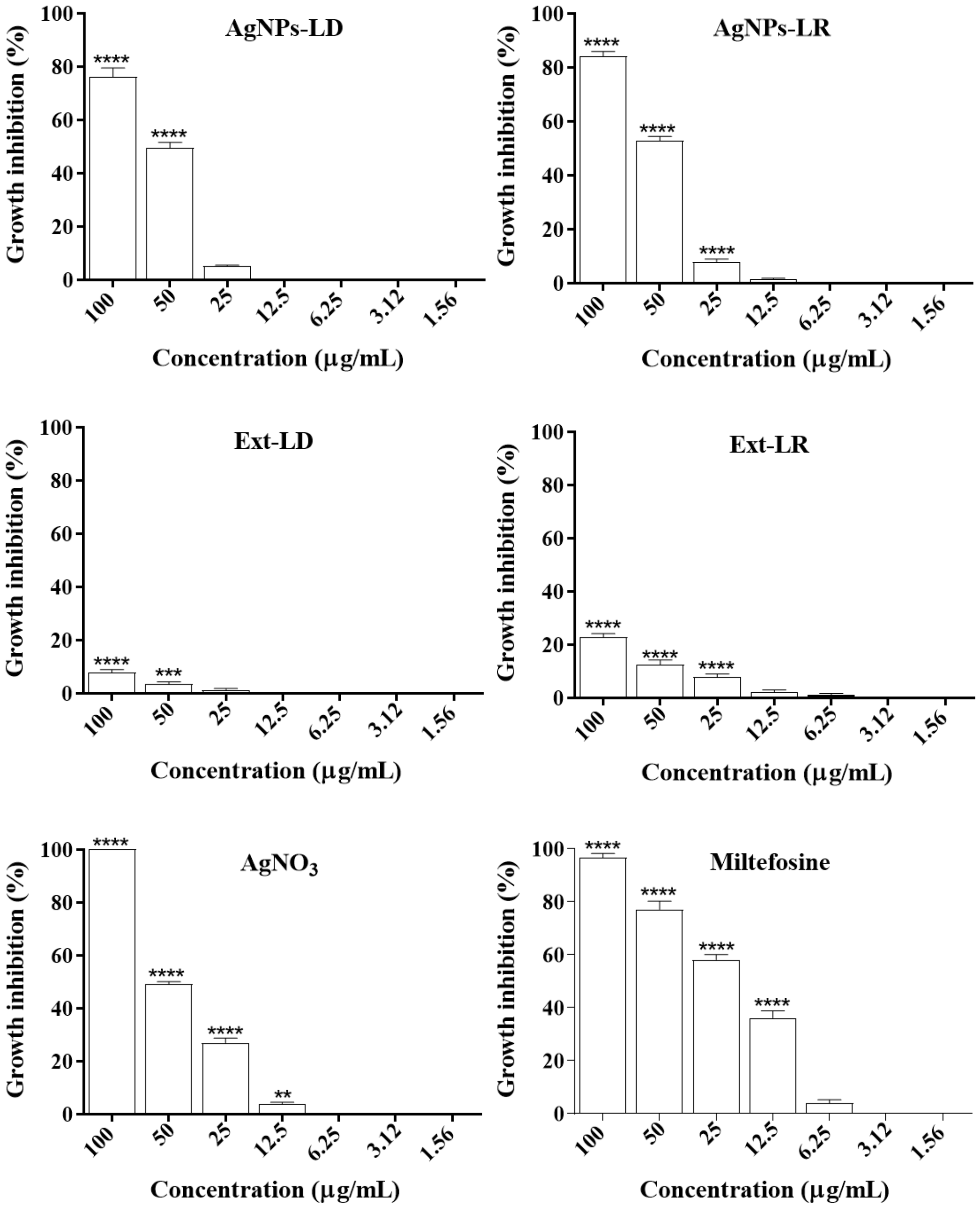

Supplementary Figure S3: Cytotoxicity of AgNPs, *Paullinia cupana* leaf extracts, aqueous $AgNO_3$ solution and miltefosine against RAW 264.7 macrophages after 72 h using the MTT colorimetric method.

The graph represents the mean ± standard deviation of the mean of three independent experiments, considering the control (0.5% DMSO in complete DMEM medium) as 100% cell viability. One-way ANOVA followed by the Bonferroni post-test was carried out to compare the groups, with *p<0.05; **p<0.01; ***p<0.001 and **** p<0.0001 compared to the control.

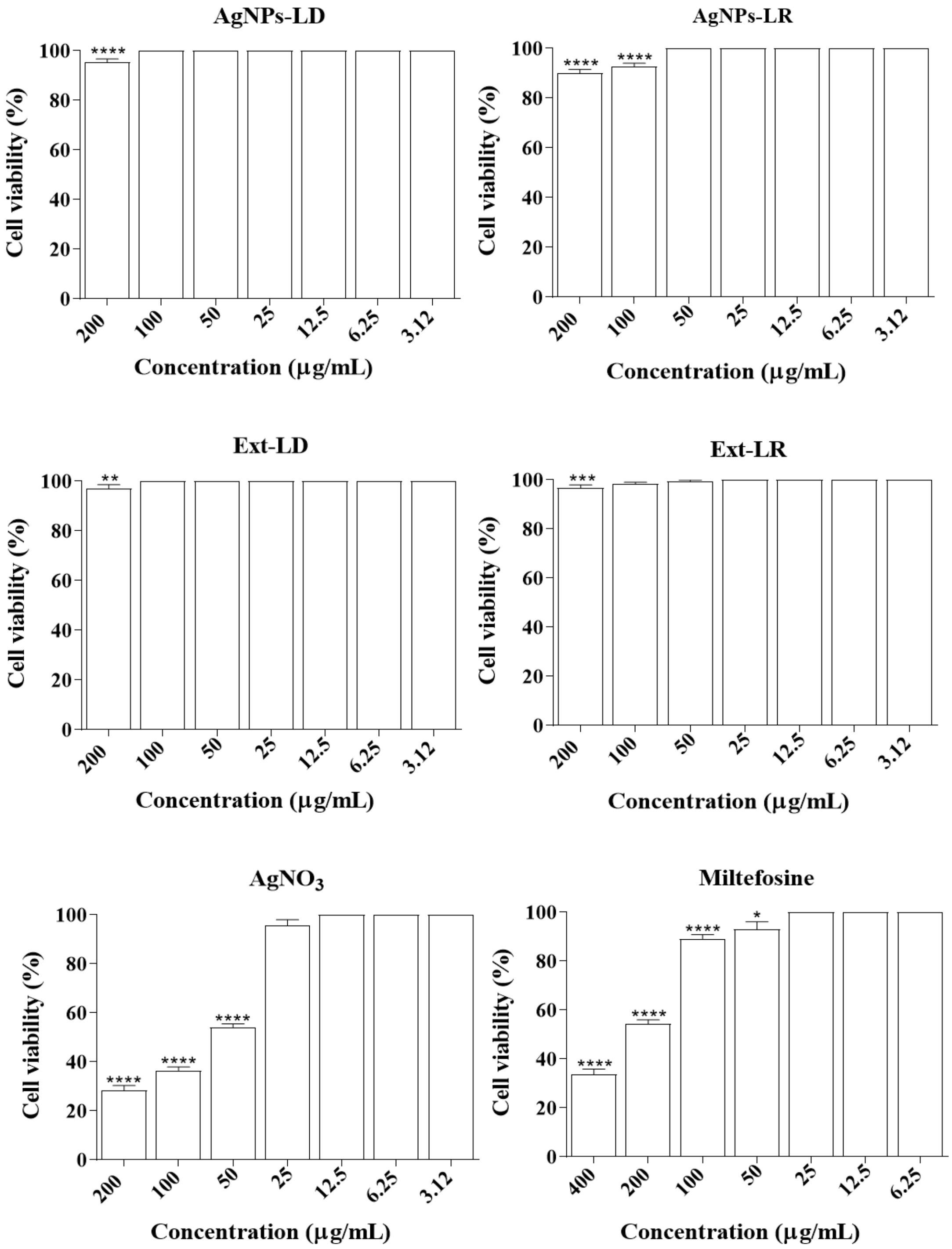

Supplementary Figure S4: (A) Dose-response curve (mortality - %) of *Aedes aegypti* larvae after exposure for 24, 48 and 72 h to different concentrations of AgNPs-LD. (B) Dose-response curve (mortality - %) of *Aedes aegypti* larvae after exposure for 24, 48 and 72 h to different concentrations of $AgNO_3$.

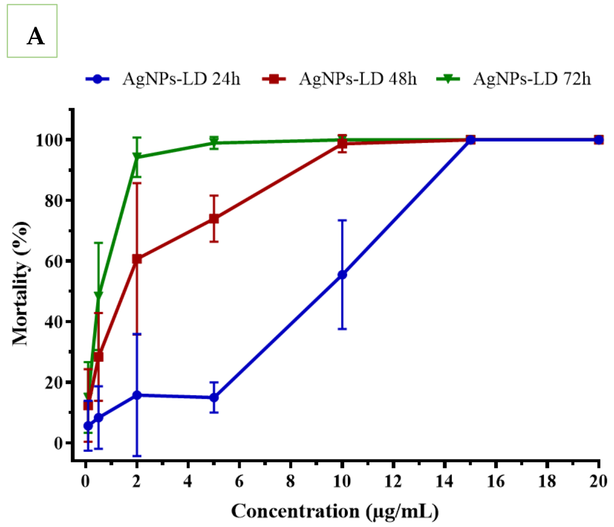

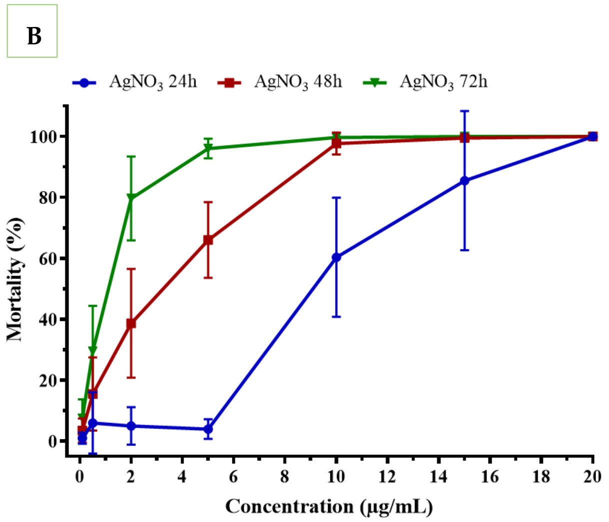

Supplementary Figure S5: (A) Dose-response curve (mortality - %) of *Aedes aegypti* pupae after exposure for 24 and 48 h to different concentrations of AgNPs-LD. (B) Dose-response curve (mortality - %) of *Aedes aegypti* pupae after exposure for 24 and 48 h to different concentrations of $AgNO_3$.

A

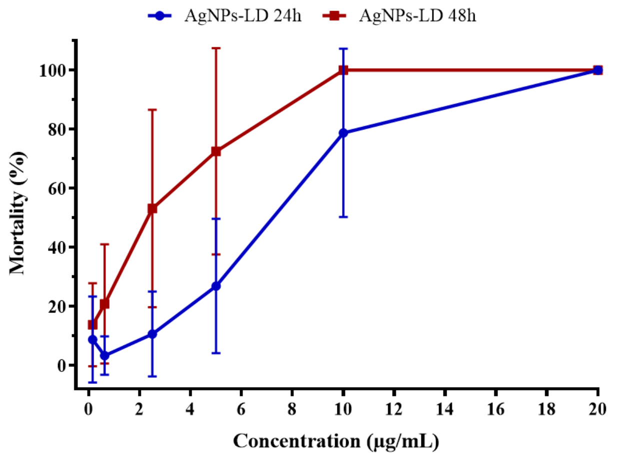

B

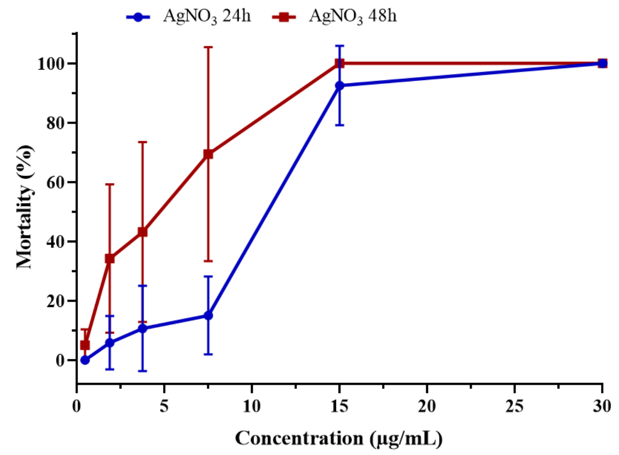

Supplementary Figure S6: (A) Removal (%) of the methylene blue (MB) dye at different exposure times to the nanocatalysts; (B) Second-order kinetic graph of the linear regression of the logarithm of absorbance *versus* time (minutes) of MB degradation in the presence of AgNPs-LD; (C) First-order kinetic graph of the linear regression of the logarithm of absorbance *versus* time (minutes) of MB degradation in the presence of AgNPs-LR.

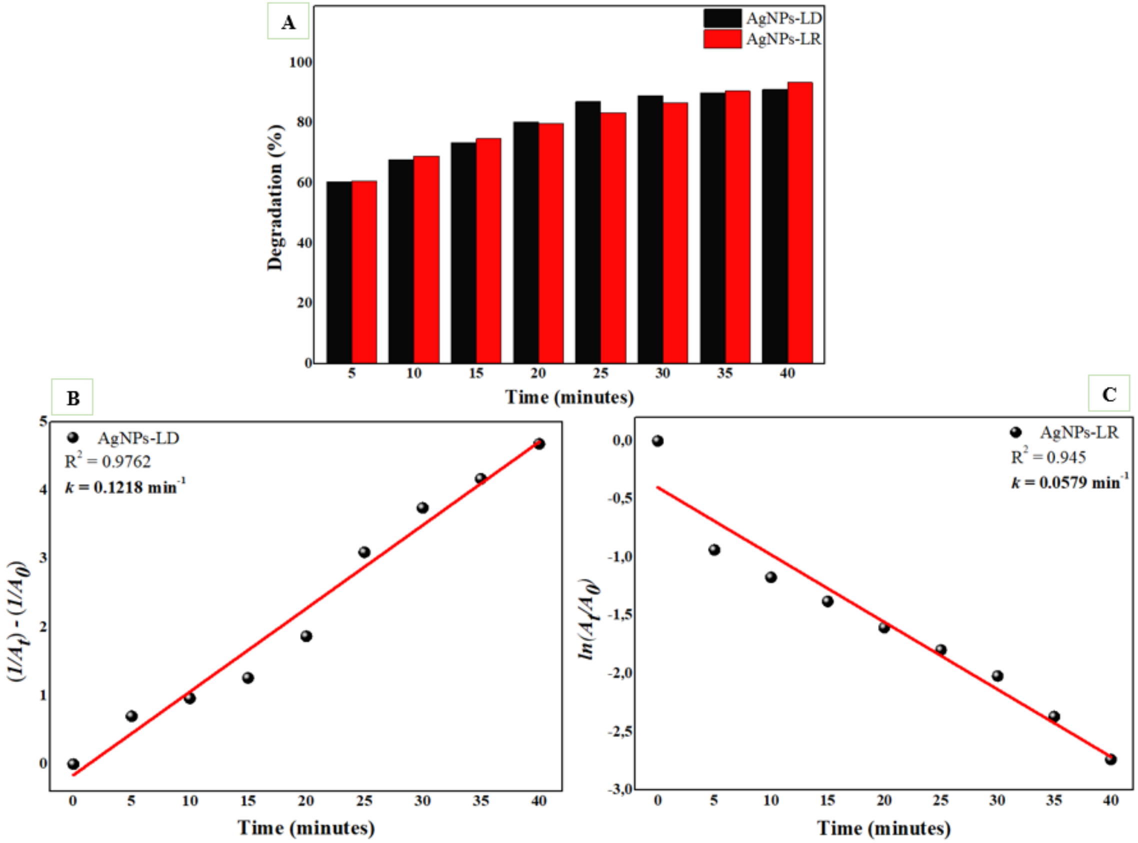

Supplementary Figure S7: (A) Removal (%) of methyl orange (MO) dye at different exposure times to the nanocatalysts; (B) Zero-order kinetic graph of the linear regression of the logarithm of absorbance *versus* time (minutes) of MO degradation in the presence of AgNPs-LD; (C) Zero-order kinetic graph of the linear regression of the logarithm of absorbance *versus* time (minutes) of MO degradation in the presence of AgNPs-LR.

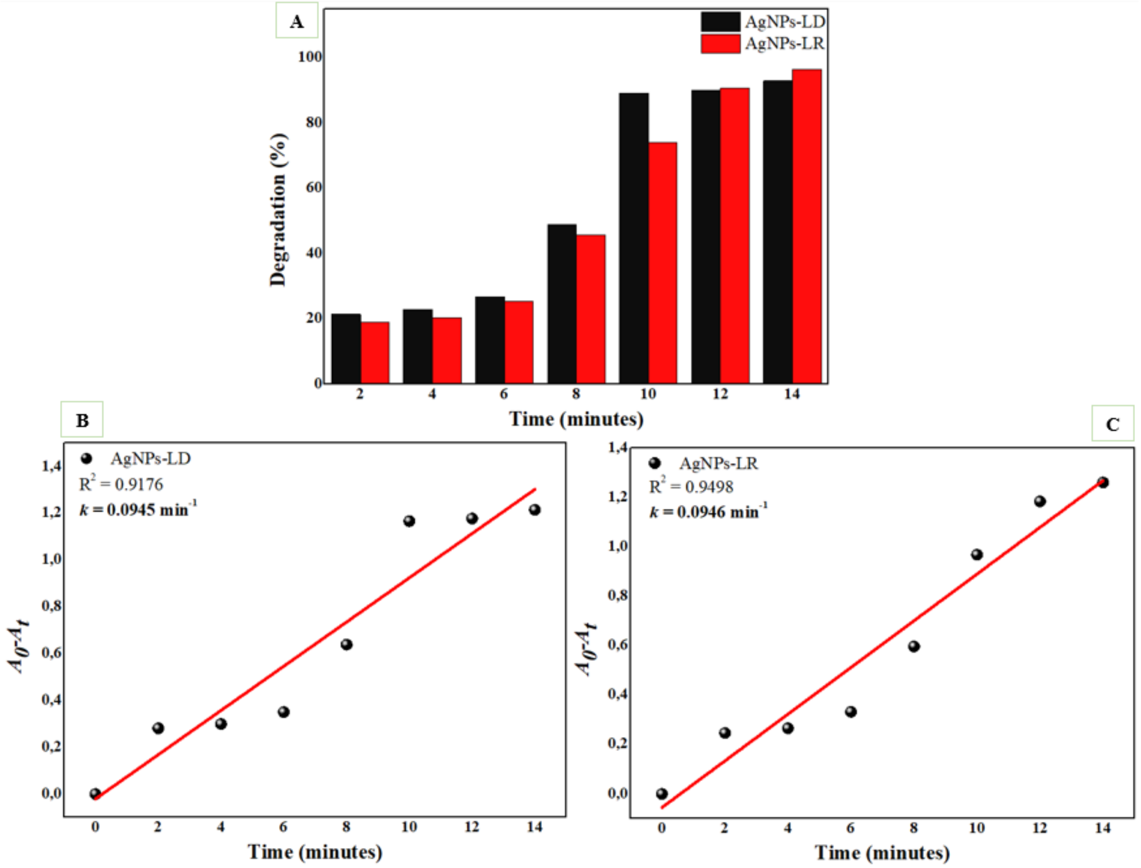

Supplement: Supplementary file 1 [file pharmaceutics-17-00356-s001.zip › pharmaceutics-3494955-supplementary.pdf]
